# p38 promoted retinal micro-angiogenesis through up-regulated RUNX1 expression in diabetic retinopathy

**DOI:** 10.1042/BSR20193256

**Published:** 2020-05-05

**Authors:** Wenjun Zou, Zhengwei Zhang, Shasha Luo, Libo Cheng, Xiaoli Huang, Nannan Ding, Jinjin Yu, Ying Pan, Zhifeng Wu

**Affiliations:** 1 Department of Ophthalmology, The Affiliated Wuxi No. 2 People’s Hospital of Nanjing Medical University, Zhongshan Road 68, Wuxi 214002, Jiangsu, China; 2 The First People’s Hospital of Lianyungang, Lianyungang 222061, Jiangsu, China

**Keywords:** angiogenesis, Diabetic retinopathy, p38, RUNX1

## Abstract

Diabetic retinopathy (DR) is the most common microvascular complication of diabetes and is characterized by visible microvascular alterations including retinal ischemia–reperfusion injury, inflammation, abnormal permeability, neovascularization and macular edema. Despite the available treatments, some patients present late in the course of the disease when treatment is more difficult. Hence, it is crucial that the new targets are found and utilized in the clinical therapy of DR. In the present study, we constructed a DR animal model and a model in HRMECs to investigate the relationship between p38 and RUNX1 in retinal micro-angiogenesis in diabetic retinopathy. We found that p38 could promote retinal micro-angiogenesis by up-regulating RUNX1 expression in diabetic retinopathy. This suggested that the p38/ RUNX1 pathway could become a new retinal micro-angiogenesis target in DR treatment.

## Introduction

Diabetic retinopathy (DR) is the most common microvascular complication of diabetes and remains as a leading cause of vision loss in adults [1–3]. There are no obvious early symptoms in diabetic retinopathy [2]; hence, when visual problems begin, retinopathy has already advanced to almost a point of no return [4]. Laser therapy is the mainly effective therapy for preservation of sight in proliferative retinopathy [5]; however, it is not effective for reversing visual loss [5]. DR is a progressive disease characterized by visible microvascular alterations, including retinal ischemia–reperfusion injury, inflammation, abnormal permeability, neovascularization and macular edema [6–8]. Hence, it is of great importance to investigate the potential molecular mechanism of retinal neovascularization to developing effective diagnosis and therapy strategies for DR patients.

The mitogen-activated protein kinase (MAPK)/p38 signaling pathway is well known to exert a crucial role in balancing cell survival and cell death [9–11]. Inhibition of the MAPK/p38 signaling pathway is known to suppress cell apoptosis and the expression of pro-inflammatory cytokines in related cells [12,13]. Related studies report that targeting the VEGF/HIP-1/p38 signaling pathway can regulate pathophysiological angiogenesis and tissue repair [14,15].

Runt-related transcription factors (RUNXs) are essential regulators of proliferation and differentiation in cells in metazoans [16–18]. There are three metazoan RUNXs (RUNX1, RUNX2 and RUNX3) with different tissue-specific gene expression and functions [18]. RUNX3 plays a crucial role in definitive hematopoiesis, differentiation of T- and B-cell lineages, and neuronal development [19,20]. Recent report shows that RUNX1 may be a putative molecular target of therapies against glioma metastasis and angiogenesis that function through the activation of the p38 MAPK signaling pathway [21]. However, the relationship between p38 and RUNX1 in retinal micro-angiogenesis in diabetic retinopathy is still unknown.

In the present study, we constructed a DR animal model and a model in HRMECs to investigate the relationship between p38 and RUNX1 in retinal micro-angiogenesis in diabetic retinopathy. We found that p38 could promote retinal micro-angiogenesis by up-regulating RUNX1 expression in diabetic retinopathy. This suggested that the p38/ RUNX1 pathway could become a new retinal micro-angiogenesis target in DR treatment.

## Materials and methods

### Cell culture and stimulation

The primary human retinal microvascular endothelial cells (HRMECs) was purchased from Cell Systems (Kirkland, WA, U.S.A.), and routinely cultured in M199 medium (Millipore, Temecula, CA) supplemented with 100 units of penicillin and 100 μg of streptomycin per milliliter of medium. All cells (passages 5–12) were cultured in plastic-ware and maintained in an atmosphere of 5% CO_2_ at 37°C.

### Construction and treatment of high glucose cell model

The high glucose cell model was constructed as described [22,23]. Briefly, HRMECs were cultured in conditioned medium with 5 mM (serving as the normal glucose (NG) group) or 30 mM (HG group) d-glucose (Sigma, Darmstadt, Germany) and incubated at 37°C with 5% CO_2_, to control osmotic pressure, 25 mM mannose was added to the medium. Then, the HG group was treated with or without 10 μM p38 inhibitor SB203580 (MedChemExpress, New Jersey, U.S.A.) or 50 ng/ml p38 agonist Anisomycin (MedChemExpress, New Jersey, U.S.A.) for 24 h. Each inhibitor or agonist was dissolved in dimethyl sulfoxide (DMSO) to a 50 mM concentration for use as stock solutions that were diluted to the required concentrations for *in vitro* studies.

In another experiment, the HRMECs were treated with or without p38 agonist for 24 h, and then with or without RUNX1 adenovirus (sh- RUNX1) or sh-NC for 12 h. Then, the cells were used in Western blotting and tube formation experiments.

### Constructive and treatment of the DR model

Male C56BL/6J mice (10 weeks old) were obtained from Yangzhou University (Certificate of quality No. SCXK (su) 2017-0007) and housed under standard conditions at the Animal Research Center of China Pharmaceutical University. All animal experiments were performed at the Animal Research Center of China Pharmaceutical University, according to protocols approved by the Ethics Committee of China Pharmaceutical University (Approval No. SYXK (su) 2016-0011). The mice were divided into two groups: normal group and hyperglycemic (HG) group. Streptozotocin (STZ)-induced hyperglycemic mice were utilized as Type I diabetic-like model associated with retinopathy (Wang et al., 2019b). Male C57BL/6J mice were received one time/day constitutive intraperitoneal injections of 50 mg/kg STZ in a citric buffer (pH 4.5) for 5 days. After last injection, the 4-h fasting blood glucose level was determined, and the fasting blood glucose levels were within 15.0–20.0 nmol/l. All animals were killed by cervical dislocation, and the eyeballs were collected for analysis. Some retinas were enucleated and placed in 4% paraformaldehyde overnight for immunofluorescence analysis. Other retinas were reserved at −80°C for Western blotting experiments. No anesthetics were used in this model.

### Membrane and nuclear protein extraction

The nucleoprotein and membrane proteins from HRMECs and tissue samples were extracted by using Membrane Nuclear and Cytoplasmic Protein Extraction Kit (Sangon Biotech, Shanghai, China). All proteins were detected by Western blotting.

### Western blot analysis

For Western blot analysis, HRMECs and tissue samples were lysed in RIPA buffer containing protease inhibitor cocktail (Generay, Shanghai, China). The proteins were transferred to PVDF membranes and probed with primary antibodies, including anti-RUNX1 (#ab35962; dilution, 1:1000), anti-p38 (#ab170099; dilution, 1:1000), anti-VEGF (#ab222510; dilution, 1:1000) and anti-GAPDH (#ab181602; dilution, 1:1000) or anti-H3 (#ab1791; dilution, 1:1000); all primary antibodies were purchased from Abcam (Cambridge, MA, U.S.A.). The second antibody HRP-conjugated goat anti-mouse (#ab19195; dilution, 1:10,000) and goat anti-rabbit IgG (#ab6721; dilution, 1:10,000) were used to detect the expression of T-RUNX1, T-p38, T-VEGF and T-GAPDH, respectively; all secondary antibodies were purchase from Abcam (Cambridge, MA, U.S.A.). The blots were detected using Bio-Imaging System and Quality One 1-D analysis software (Bio-Rad, Richmond, CA, U.S.A.).

### Immunofluorescence staining

The cryo-sections from mouse retina tissues were fixed with ice-cold acetone for 20 min. The slides were blocked with 5% BSA in PBS for 1 h and subjected to incubation at 4°C overnight with biotin–anti-mouse CD31 primary antibody (#ab222783; dilution, 1:100; Abcam, Cambridge, MA, U.S.A.). Slides were washed and then incubated with streptavidin–Alexa Fluor 488 conjugate (#ab150073; dilution, 1:200; Abcam, Cambridge, MA, U.S.A.) for 90 min. The slides were costained with DAPI and mounted with fluoro-gel (Electron Microscopy Science). Confocal images were acquired by Leica TCS SP5 confocal microscope system (Leica Microsystems, Germany) and quantified by AxioVision 4.6.3.0 software (Carl Zeiss AG, Germany).

HRMECs were cultured in conditioned medium with 5 mM d-glucose (serving as the normal glucose (NG) group) or 30 mM d-glucose (the HG group). HG groups were treated with or without p38 inhibitor. Then, the cells were fixed with 10% paraformaldehyde for 10 min and washed with PBS for 5 min three times. The cell membrane was punctured with 0.5% Triton X-100 for 10 min and wash with PBS three times. Then, the cells were blocked with 3% BSA in PBS for 30 min and subjected to incubation at 4°C overnight with biotin–anti-human RUNX1 primary antibody (#ab23980; dilution, 1:100; Abcam, Cambridge, MA, U.S.A.). Next, the cells were washed with 0.35% Tween 20 three times, and incubated with streptavidin–Alexa Fluor 488 conjugate (1:400) for 1 h. the cells were washed with 0.35% Tween 20 three times, and incubated with DAPI (100 ng/ml) for 10 min. Last, the cells were washed with 0.35% Tween 20 three times. Confocal images were acquired by Leica TCS SP5 confocal microscope system (Leica Microsystems, Germany) and quantified by AxioVision 4.6.3.0 (Carl Zeiss AG, Germany).

### Retinal microvascular endothelial cell tube formation assays

The Matrigel (100 μl per well) was evenly spread on the bottom of a 24-well plate and allowed to solidify at 37°C with 5% CO_2_ for 2 h. Different treated HRMECs were digested and centrifuged at 1000 ***g***; the supernatant was removed and the cells were suspended in complete medium; the cells were separately seeded in 24-well plates at 3 × 10^5^ cells per well and cultured at 37°C with 5% CO_2_ for 8 h. The tube formation of retinal microvascular endothelial cells was photographed and analyzed by the Angiogenesis Analyzer Plugin of ImageJ software.

### Measurements of VEGF

Retinal tissue from the control group and DR model group was added to PBS and homogenized; the homogenate was centrifuged at 12,000 ***g*** at 4°C for 10 min. Then, the supernatant was tested for VEGF by mouse VEGF ELISA kit (R&D Systems, MN, U.S.A.). The cells experiments, the cells culture supernatants from the different treatment groups were harvested and tested using mouse VEGF ELISA kit.

### Statistical analysis

The data were statistically analyzed by Student’s *t*-test using Graph pad prism 4.0 (Graph pad Software, La Jolla, CA). *P* < 0.05 was considered statistically significant.

## Results

### RUNX1 was highly expressed in diabetic retinopathy

**Figure 1:**
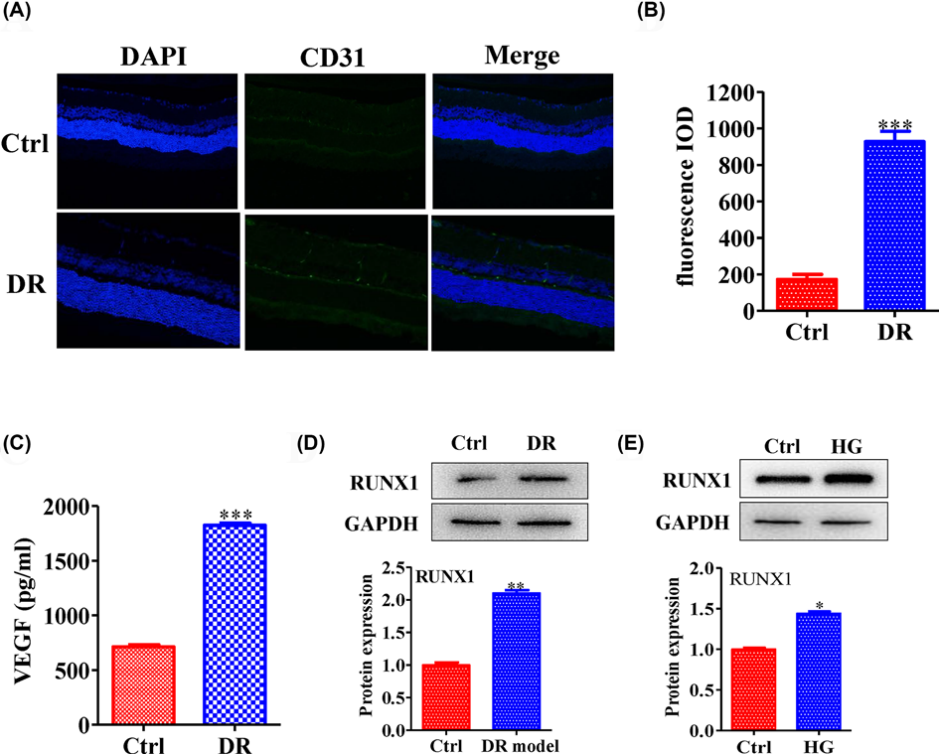
RUNX1 was highly expressed in diabetic retinopathy (**A**) The expression of the vascular marker CD31 was increased in the DR group by confocal detection. (**B**) Quantification of the fluorescence signal was showed. Meanwhile, (**C**) the VEGF secretion levels in the DR group were increased compared with those in the control group. Moreover, Western blotting was performed, and RUNX1 expression levels were quantified. The results showed that RUNX1 was highly expressed in (**D**) the DR group and (**E**) the HG group. All experiments were performed triplicate. Values are expressed as the mean ± SD (*P<0.05, **P<0.01 and ***P<0.001 compared with the control).

Some reports show that the inhibition of RUNX1 activity results in the reduction of neovascular tufts in retinopathy [24], suggesting that RUNX1 has a close relationship with angiogenesis. Hence, we constructed DR model to investigate the expression level of RUNX1 and level of angiogenesis. The results showed that the expression of vascular marker CD31 was increased in DR group by confocal detection (Figure 1A,B) (Fluorescence IOD: Ctrl vs DR group: 172.67 ± 48.42 vs 927.33 ± 99.55); meanwhile, the VEGF secretion levels in DR group were increased compared with those in the control group (Figure 1C) (Ctrl vs DR group: 711.8 ± 36.88 vs 1823.67 ± 32.99). Moreover, we utilized Western blotting to measure the expression of RUNX1 in the DR model and HG cell model. The results suggested that RUNX1 level was higher in the DR model and HG cell model compared with normal group (Figure 1D,E) (quantification of WB: Ctrl vs DR group: 1 ± 0.06 vs 2.1 ± 0.07) (Ctrl vs HG group: 1 ± 0.03 vs 1.44 ± 0.03). All of these results suggested that RUNX1 was highly expressed in diabetic retinopathy.

### The angiogenesis level was inhibited by silencing RUNX1

**Figure 2:**
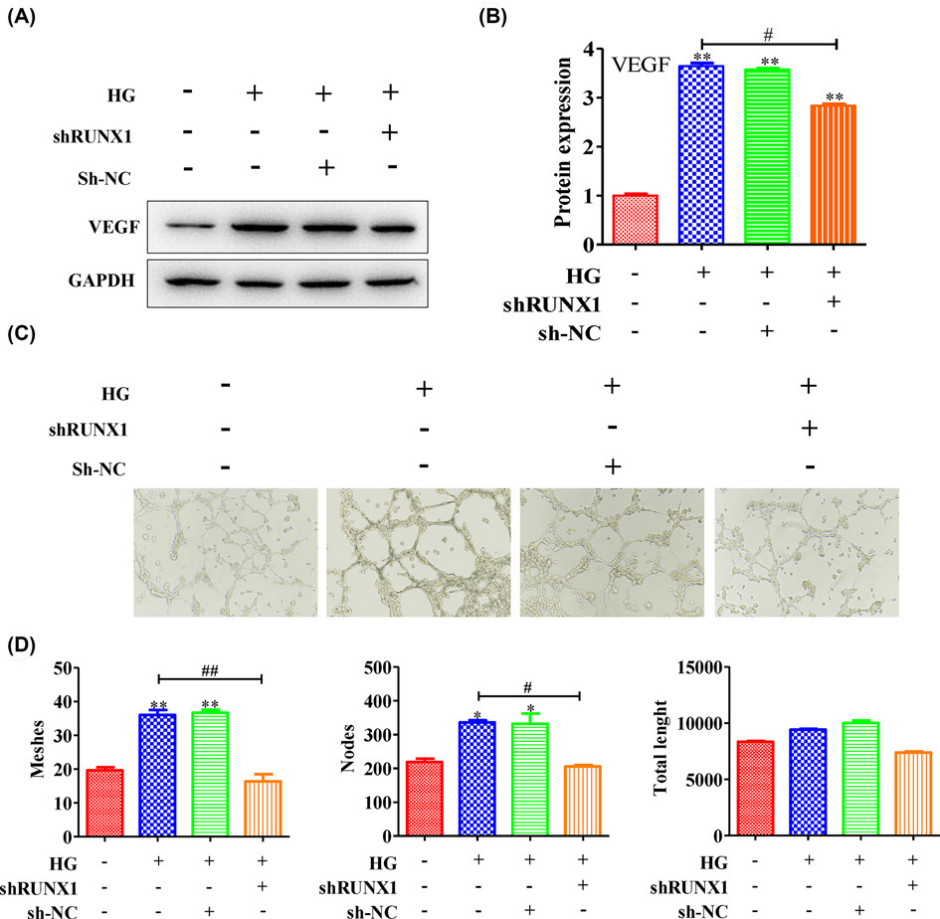
The angiogenesis level was inhibited by silencing RUNX1 HRMECs were stimulated with or without high levels of glucose and then treated with or without RUNX1 adenovirus. (**A**) The expression level of VEGF was measured, and (**B**) the quantification was showed. Meanwhile, (**C**) the tubes that formed were photographed. (**D**) The number of tubes, nodes and the tube length were determined. All experiments were performed triplicate. Data are presented as the mean ± standard deviation from triplicate wells; *P<0.05 and **P<0.01 compared with the control; ^#^*P*<0.05 and ^##^*P*<0.01 compared with HG-induced HRMEC cell group.

The above data suggested that RUNX1 was highly expressed in diabetic retinopathy [24]. Hence, we utilized RUNX1 adenovirus to silence its expression in HG cell model. The results showed that high glucose could enhance the VEGF protein expression level, and its expression level was inhibited by silencing RUNX1 (Figure 2A,B) (quantification of WB: Ctrl vs HG group: 1 ± 0.03 vs 3.64 ± 0.11; HG group vs shRUNX1 group: 3.64 ± 0.11 vs 2.83 ± 0.05). In addition, we analyzed the tube formation level of retinal microvascular endothelial cells in HRMECs. Figure 2C showed that the tube formation level was obviously enhanced in HG HRMECs and was inhibited by silencing RUNX1. Then, the number of tubes, nodes and tube length were counted, and the results exhibited that HRMECs in high glucose medium could form more tubes and nudes than control (tube meshes: Ctrl vs HG group: 19.66 ± 1.52 vs 36 ± 2.64; nodes: 219 ± 16.64 vs 336.33 ± 10.26); meanwhile, the total tube length in HG group was higher than control group (total length: Ctrl vs HG group: 8327 ± 112.6 vs 9418 ± 106.15); however, after silencing RUNX1, the tube meshes, nudes and tube length were reduced comparing with HG group (Figure 2D) (tube meshes: HG group vs shRUNX1 group: 36 ± 2.64 vs 16.33 ± 3.78; nodes: 336.33 ± 10.26 vs 205.66 ± 7.57; total length: 9418 ± 106.15 vs 7376.66 ± 173.78). These result suggested that the angiogenesis level was inhibited through silencing RUNX1.

### RUNX1 expression level was down-regulated by inhibiting p38 expression

**Figure 3:**
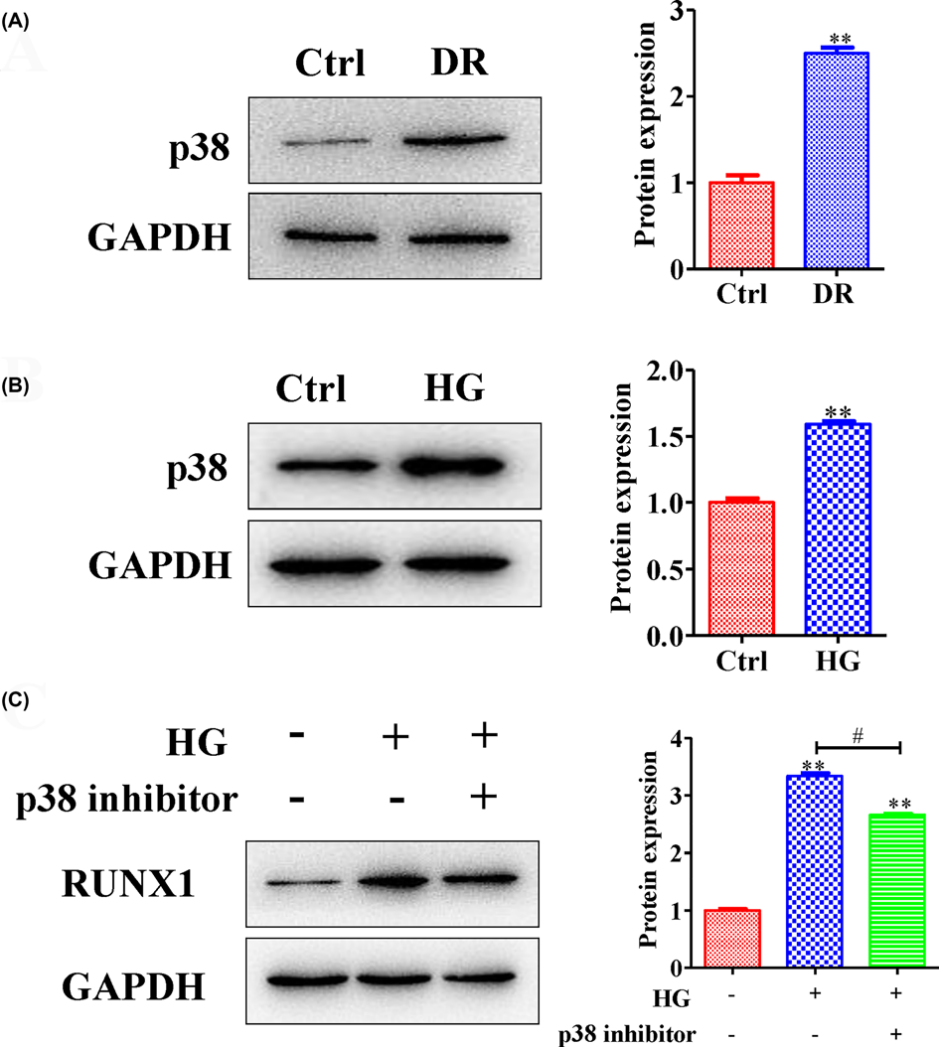
The RUNX1 expression level was down-regulated by inhibiting p38 expression The p38 expression level was measured by Western blotting; meanwhile, the results of the Western blot experiments are shown. The results showed that p38 was highly expressed in (**A**) the DR group and (**B**) the HG group. (**C**) After inhibiting p38, the expression level of RUNX1 was down-regulated under high glucose conditions. All experiments were performed triplicate; **P<0.01 compared with the control; ^#^*P*<0.05 compared with the HG-induced HRMECs group.

Recent report shows that RUNX1 may be a putative molecular target of therapies against glioma metastasis and angiogenesis through the activation of p38 MAPK signaling pathway [21]. We measured the expression level of p38 in DR model and HG cell model. The results showed that p38 protein was highly expressed in DR model (Figure 3A,B) (quantification of WB: Ctrl vs DR group: 1 ± 0.14 vs 2.49 ± 0.11; Ctrl vs HG group: 1 ± 0.05 vs 1.59 ± 0.03). Then, the relationship between RUNX1 and p38 was investigated, and the results showed that RUNX1 expression level was down-regulated by inhibiting p38 expression (Figure 3C) (quantification of WB: Ctrl vs HG group vs p38 inhibitor group: 1 ± 0.04 vs 3.33 ± 0.09 vs 2.66 ± 0.04). Taken together, these data suggested that p38 could regulate RUNX1 expression in diabetic retinopathy.

### High levels of glucose caused RUNX1 to enter the nucleus via p38 activation

**Figure 4:**
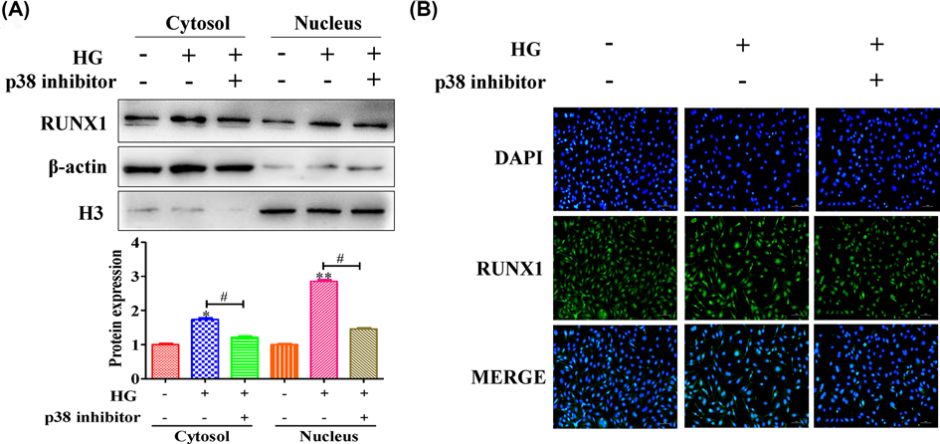
High glucose levels caused RUNX1 to enter the nucleus by activating p38 (**A**) The ability of RUNX1 to enter the nucleus was investigated, and quantification of the results was showed. The results showed that high glucose levels caused RUNX1 to transition from the cytoplasm to the nucleus. (**B**) The immunofluorescence results showed that high glucose levels enhanced RUNX1 transition from the cytoplasm to the nucleus. However, this transition was blocked by inhibited p38. All experiments were performed triplicate; *P<0.05 and **P<0.01 compared with the control; ^#^*P*<0.05 compared with the HG-induced HRMECs group.

RUNX1, an important transcription factor, is also involved in embryonic development, tumorigenesis, the immune response and, especially, the inflammatory response. We investigated the ability of RUNX1 to enter the nucleus. The results showed that the protein expression level of RUNX1 was enhanced in the cytoplasm (Figure 4A) (quantification of WB (Cytosol): Ctrl vs HG group: 1 ± 0.05 vs 1.74 ± 0.07), while the protein expression level was up-regulated in the nucleus (Figure 4A) (quantification of WB (Nucleus): Ctrl vs HG group: 1 ± 0.03 vs 2.85 ± 0.07); however, after inhibiting p38 expression, the protein expression level of RUNX1 was down-regulated in the cytoplasm and nucleus (Figure 4A) (quantification of WB (Cytosol): HG group vs p38 inhibitor group: 1.74 ± 0.07 vs 1.21 ± 0.04) (quantification of WB (Nucleus): HG group vs p38 inhibitor group: 2.85 ± 0.07 vs 1.46 ± 0.04). In addition, the immunofluorescence results showed that high glucose levels enhanced RUNX1 transition from the cytoplasm to the nucleus; but, this transition was blocked by inhibiting p38 (Figure 4B). These data suggested that high glucose levels caused RUNX1 to enter the nucleus by activating p38.

### High glucose levels caused RUNX1-dependent abnormal vascular proliferation via p38 activation

**Figure 5:**
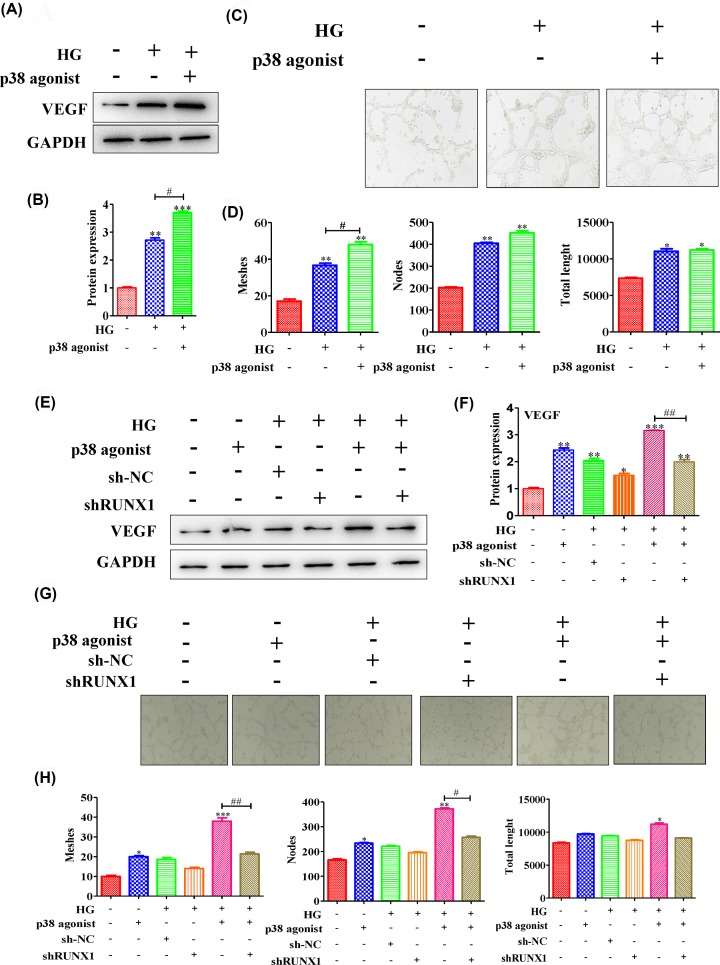
High glucose levels caused RUNX1-dependent abnormal vascular proliferation by activating p38 (**A**) The expression levels of RUNX1 from HRMECs treated with or without p38 agonist were measured in normal or high glucose conditions, and (**B**) the quantification was showed; meanwhile, (**C**) the tube formation was photographed; then, (**D**) the number of tubes, nodes and tube length were determined. (**E**) The RUNX1 protein expression level was measured by Western blotting in normal or HG cells with or without treatment with p38 agonist or sh-RUNX1 adenovirus. (**F**) The quantification was showed. (**G**) The tubes that formed were photographed. (**H**) The number of tubes, nodes and tube length were counted. All experiments were performed triplicate; *P<0.05 and **P<0.01 compared with the control; ^#^*P*<0.05 compared with HG-induced HRMECs group or the HG + p38 induced group.

Studies report that targeting to the VEGF/HIP-1/p38 signaling pathway can regulate pathophysiological angiogenesis and tissue repair. Hence, we investigated the relationship between p38 and angiogenesis. The results showed that high glucose levels enhanced VEGF expression; the protein expression level was up-regulated after stimulation with p38 agonist (Figure 5A,B) (quantification of WB: Ctrl vs HG group vs p38 agonist group: 1 ± 0.05 vs 2.71 ± 0.13 vs 3.7 ± 0.08). Then, the tube formation level was observed; and the results showed that tube formation level was obviously increased in HG HRMECs via activation of p38 (Figure 5C). The number of tubes, nodes and tube length were determined, and the results exhibited that HRMECs in high glucose could form more tubes and nodes than control cells (Figure 5D) (tube meshes: Ctrl vs HG group vs p38 agonist group: 17 ± 2 vs 36.66 ± 2.08 *vs* 48 ± 2.64; nodes: 202.66 ± 6.11 vs 404.66 ± 7.57 vs 452.66 ± 16.65); the tube total length in the HG group was higher than that in the control group; after activating p38, the number of tube, nodes and tube length were enhanced compared with those in the HG group (Figure 5D) (total length: Ctrl vs HG group vs p38 agonist group: 7352 ± 182.07 vs 11028.67 ± 605.46 vs 11205.33 ± 314.83). Next, we further investigated the relationship between p38-mediated RUNX1 and vascular proliferation. The results illustrated that activation of p38 or high glucose could up-regulated VEGF expression level in high glucose conditions (quantification of WB: Ctrl vs p38 agonist group vs HG group: 1 ± 0.06 vs 2.43 ± 0.12 vs 2.05 ± 0.12), and silencing RUNX1 could decrease the protein expression level in high glucose conditions (Figure 5E,F) (quantification of WB: HG group vs HG + shRUNX1 group: 2.05 ± 0.12 vs 1.49 ± 0.11). However, after simultaneously activating p38 and silencing RUNX1, VEGF expression levels were decreased in high glucose conditions (Figure 5 E,F) (p38 agonist group (HG condition) vs p38 agonist + shRUNX1 group (HG condition): 3.16 ± 0.01 vs 1.99 ± 0.14).

Moreover, the tube formation level was obviously increased in HG HRMECs via p38 activation and was inhibited by silencing RUNX1 (Figure 5G). The number of tube, nodes and tube length were determined, and the results exhibited that activating p38 led to the formation of more tubes and nodes than were seen in control HRMECs in high glucose conditions (Figure 5H) (tube meshes: Ctrl vs p38 agonist group vs HG + sh-NC group vs HG + p38 agonist group: 10 ± 1 vs 20 ± 1 vs 18.66 ± 1.52 vs 38 ± 3; nodes: 166.33 ± 7.02 vs 233.66 ± 5.5 vs 220.66 ± 6.42 vs 372 ± 7); the total tube length in the HG group was higher than that in the control group because of the activation of p38 in the HG group (total lenght: Ctrl vs p38 agonist group vs HG + sh-NC group vs HG + p38 agonist group: 8378 ± 155.15 vs 9735.33 ± 87.76 vs 9423 ± 102.5 vs 11210 ± 332.58). After simultaneously activating p38 and silencing RUNX1, the number of tube, nodes and tube length were decreased (Figure 5H) (tube meshes: HG + sh-NC group vs HG + shRUNX1 group: 18.66 ± 1.52 vs 14 ± 1; HG + p38 agonist group vs HG + p38 agonist + shRUNX1 group: 38 ± 3 vs 21.33 ± 1.52; nodes: HG + sh-NC group vs HG + shRUNX1 group: 220.66 ± 6.42 vs 195 ± 6.55; HG + p38 agonist group vs HG + p38 agonist + shRUNX1 group: 372 ± 7 vs 257.33 ± 8.5; total length: HG + sh-NC group vs HG + shRUNX1 group: 9423 ± 102.5 vs 8767 ± 167.24; HG + p38 agonist group vs HG + p38 agonist + shRUNX1 group: 11210 ± 332.58 vs 9109.66 ± 91.35). All of these data suggested that high glucose levels caused RUNX1-dependent abnormal vascular proliferation via p38 activation.

## Discussion

DR is a common complication of diabetes mellitus, and the underlying mechanisms are still not fully delineated [3,25]. DR is induced by diabetes, which causes the pathology of retinal capillaries, arterioles and venules and subsequent leakage from or occlusion of small vessels [14,25,26]. Hence, it is still necessary to find a new mechanism to improve therapies for DR.

In the present study, we constructed a DR animal model and a high model in HRMECs to investigate the relationship between p38 and RUNX1 in retinal micro-angiogenesis in diabetic retinopathy. The results showed that p38 and RUNX1 play crucial roles in regulating retinal micro-angiogenesis in DR; p38 could mediate RUNX1 expression to promote angiogenesis. This suggested that the p38/ RUNX1 pathway could become a new retinal micro-angiogenesis target in DR treatment.

Multiple extracellular stimuli can activate the MAPK signaling pathway, including stress induced from high glucose levels [27,28]. Activation of the MAPK signaling pathway can cause a cascade of physiological outcome, such as apoptosis, proliferation, cell mitosis and transcription of certain genes [29]. Related reports have shown that some small RNAs can effectively improve diabetic retinopathy by regulating the expression level of phosphorylated p38-MAPK but not that of phosphorylated ERK or JNK [9]. In the present study, our results suggested that p38 protein level was highly expressed in the DR model (Figure 3A,B); p38 enhanced tube formation in HG HRMECs (Figure 5C,D), which suggested that p38 played a crucial role in the regulation of DR.

RUNX1, an important transcription factor, is also involved in embryonic development, tumorigenesis, the immune response and, especially, the inflammatory response [30–32]. Some reports show that inhibition of RUNX1 activity with the small molecule resulted in a significant reduction in neovascular tufts in oxygen-induced retinopathy, supporting the feasibility of targeting RUNX1 in aberrant retinal angiogenesis [24]. In the present study, we found that RUNX1 was highly expressed in diabetic retinopathy (Figure 1A,B), and the angiogenesis level was inhibited by silencing RUNX1 (Figure 2A–D). Moreover, the RUNX1 expression level was down-regulated by inhibiting p38 expression, and high glucose levels caused RUNX1 to enter the nucleus via p38 activation. In addition, high glucose levels caused RUNX1-dependent abnormal vascular proliferation by activating p38. Hence, taken together, these results suggested that p38 promoted retinal micro-angiogenesis by up-regulated RUNX1 expression in diabetic retinopathy.

## Conclusions

In summary, we found that RUNX1 was highly expressed in DR, and angiogenesis and tube formation levels were inhibited by silencing RUNX1. Moreover, we found that p38 and RUNX1 had a close relationship in mediating angiogenesis in DR. High glucose levels caused RUNX1 to enter the nucleus via p38 activation, while high glucose levels caused RUNX1-dependent abnormal vascular proliferation via p38 activation. In addition, the tube formation level was obviously increased in HG HRMECs via p38 activation and was inhibited by silencing of RUNX1; p38 activation leads to an increased number of tubes, nodes than were seen in control HRMECs in high glucose condition, and the total tube length was higher in the HG group than in the control. After simultaneously activating p38 and silencing RUNX1, the number of tubes, nodes and tube length was decreased. These data showed that the p38/RUNX1 signaling pathway is crucial in mediating angiogenesis in DR. This suggested that the p38/ RUNX1 pathway could become a new retinal micro-angiogenesis target in DR treatment.

## Abbreviations

DR: diabetic retinopathy
HRMEC: human retinal microvascular endothelial cell
MAPK: mitogen-activated protein kinase
RUNX: Runt-related transcription factor
